# Mercury in Pyorrhœa

**Published:** 1916-12

**Authors:** Barton L. Wright


### MERCURY IN PYORRHŒA.

BY BARTON L. WRIGHT, M.D.

The following case well illustrates many of the points in treatment: Chief Master-at-Arms, U. S. Navy, age 48 10/12 years, married, wife perfectly healthy, never conceived. Admitted to the U. S. Naval Hospital, Portsmouth, N. H., from the U. S. S. Leonidas, October 1, 1914, as with chronic articular rheumatism, all joints involved, moderate temperature, suffering intensely. Treatment: bed, salicylates, bicarbonate of soda, various local applications. Wassermann negative. He remained under treatment until January 19, 1915; during this time three additional Wassermann tests were negative.

There being no change in his condition, on the latter date he was transferred to the U. S. Army and Navy General Hospital, Hot Springs, Ark.

This institution is devoted solely to the treatment of the various forms of so-called rheumatism, excepting syphilitic, patients with this disease not being accepted.

Upon admission, he was given a fifth Wassermann test, which was also negative. He remained under the special treatment given in this hospital for about four months, when he was discharged improved, all joints being normal, excepting those of both feet. Upon his arrival on board the receiving ship at the Navy Yard, Norfolk, Va., May 12, 1915, he was immediately transferred to the Naval Hospital at that place for further treatment.

His home being in Portsmouth, N. H., he requested transfer to the Navy Yard at that place, and reported on board of the U. S. S. Southery, June 2, 1915.

Fearing that if he presented himself to the medical officer, that he would again be sent to the hospital and probably surveyed and discharged from the service for physical disability, he explained to his commanding officer his condition, who kindly gave him very light duty in the enlisted men's reading room, which did not require him to be on his feet.

He continued in the performance of his duty until July 27, 1915, when he reported to me at the Navy Yard Dispensary, saying that he "was all in," giving the following history up to the time of his present illness. Had never been sick until two years prior to this time, when he had an attack of so-called acute rheumatic fever, for which he was treated in a naval hospital for some eight weeks, when he was discharged cured, and had remained so until a few days before his admission to the Naval Hospital, Portsmouth, N. H., October 1, 1914.

On July 27, 1915, inspection revealed a man five feet six inches in height, apparently well nourished, whose expression was one of suffering.

He complained of inability to walk without extreme pain in both feet extending above the ankle joints.

Upon examination, the parts presented marked swelling, redness, tenderness and increase of heat. In all of the small joints of the feet and in the ankle joints motion was markedly limited; any manipulation of the joints produced great pain.

His upper teeth had all been extracted some years previously and were replaced by a fairly good plate; eight teeth were remaining in the lower jaw, imbedded in a mass of faulty gold bridge work, which was most unhygienic, with a large amount of pus exuding from around all teeth. No additional local focus of infection could be found. Weight, 143 pounds, stripped; normal weight, 153; systolic blood pressure, 150.

He was told that he had pyorrhœa, which was probably the cause of his general condition, and I would only treat him provided he would do as directed by my dental associate, whom I was sure would insist upon the removal of the faulty bridge at least.

He stated then "that he had been treated continuously for fifteen years for pyorrhœa by a number of civilian dentists"; was very much astonished to hear that this condition was the cause of his "rheumatism," could hardly believe it, "as none of his former physicians had ever asked him about his teeth, much less looked at them." He was referred to Dr. White for dental examination, who told him the bridge must be removed and possibly all the remaining teeth might have to be extracted. He consented to our combined treatment finally. On July 30th, injection gr. 5/5 mercuric succinimide; August 6th, gr. 5/5; August 12th, bridge work removed; all eight teeth very loose; the lower right second molar, considered hopeless, was extracted. Scaling of remaining teeth commenced. August 20th, injection gr. 5/5 mercuric succinimide; August 27th, gr. 5/5; September 2d, pyorrhœa pronounced cured by Dr. White; arthritis improved. Further injections continued as follows: September 3d, gr. 5/5; September 10th, gr. 5/5; September 17th, gr. 5/5; September 24th, gr. 5/5. By this time he had markedly improved and the injections were discontinued for the time being. Graduated exercise of the feet was now commenced and gradually increased. November 3d, injections renewed, gr. 5/5 administered; this produced rather severe abdominal pain and diarrhœa. Injections continued as follows: November 10th, gr. ½; November 17th, gr. ½; November 24th, gr. ½; December 1st, gr. ½. At this time all symptoms had disappeared, motion was nearly complete in all joints and function practically restored to normal; weight, 149 pounds; systolic blood pressure, 122. Treatment discontinued.

On January 10, 1916, he was discharged from the service by expiration of enlistment, being certified by the medical officer who succeeded me on the Southery as physically qualified for re-enlistment, and was re-enlisted by him the following day. He now (February 14, 1916) weighs 158 pounds, stripped.

1. Had the pyorrhœa been cured fifteen years ago this man in all probability would never have developed arthritis.

2. When the arthritis first developed, had a search been made for a local focus of infection, and found, and the proper treatment instituted, two months would probably have been the limit of his illness.

3. Had this been so the Government would have been saved many hundreds of dollars and the man much suffering.

4. The criminal fallacy of the therapeutic test for syphilis is apparent.

5. The inefficiency of the recognized treatment (salicylates and alkalies) in these conditions is apparent.

This assertion is sustained by the eminent men using Bacelli's treatment for acute articular rheumatism, as is well shown by the following abstract of Martone's article copied in full from the *Journal American Medical Association:*

Intravenous Injection of Bichloride in Acute Rheumatism.—Martone reports five extremely severe cases in which salicylic medication failed to influence the clinical picture or *caused symptoms of intoxication so serious that it had to be discontinued.* As a last resort he turned to mercuric chlorid and gave it by intravenous injections in doses from one to five mgm., repeated every day or so, well into convalescence. All the patients given this treatment recovered, and none has shown any evidence of a return of joint trouble since. Only one was left with a slight valvular defect; in the others the endocarditis, if such existed, seemed to be completely cured. He gave ten injections in one case, beginning with two, three and four mgm., and increasing to five mgm. toward the end. In another three injections seemed to conquer the disease, but he continued with three more on alternate days to make sure of the result. In another case no benefit was apparent under salicylates for a month, and then five injections of mercuric chlorid were given and the cure was soon complete. He followed Baccelli's technic in every particular. (Italics mine.)

6. The marked curative properties of mercury in these conditions must be recognized.

7. If mercury had not been administered this man would in all probability have been discharged as incurable, becoming a navy pensioner for life, thereby costing the Government thousands of dollars, for which it would receive no return.

### CONCLUSIONS.

From my results in this disease, and in other infections previously reported, corroborated by the results reported by Souligoux, Giru and Krohl in septic infections, and by Baccelli and his followers in infectious arthritis, I am convinced that in mercury, properly administered, we have found a cure for the various infections produced by the vegetable parasites, among which pyorrhœa, as a primary focus of infection, is of the utmost importance.

The eminent men mentioned have only failed to recognize the advantage of the large doses recommended by me, hence the necessity for their frequently repeated and continued administration of this parasitotropic agent.

Shortly before the death of the lamented Ehrlich he prophesied that chemo-therapy would supplant all other forms of treatment of disease. I am confident that his prophecy will shortly be fulfilled.—*Journal Allied Societies*, June, 1916.

